# Evaluating the Feasibility and Reproducibility of a Novel Insertion Method for Modular Acetabular Ceramic Liners

**DOI:** 10.3390/bioengineering10101180

**Published:** 2023-10-11

**Authors:** Sandra Hunger, Alexander Seidler, Christian Rotsch, Christoph-Eckhard Heyde, Welf-Guntram Drossel

**Affiliations:** 1 Fraunhofer Institute for Machine Tools and Forming Technology IWU, 01187 Dresden, Germany; alexander.seidler@tu-dresden.de (A.S.); christian.rotsch@iwu.fraunhofer.de (C.R.); welf-guntram.drossel@iwu.fraunhofer.de (W.-G.D.); 2 Department of Orthopaedic, Trauma and Plastic Surgery Clinic, University of Leipzig Medical Center, 04103 Leipzig, Germany; christoph-eckhard.heyde@medizin.uni-leipzig.de; 3 Institute of Machine Elements and Machine Design, Faculty of Mechanical Science and Engineering, Dresden University of Technology, 01062 Dresden, Germany; 4 Institute for Machine Tools and Production Processes, Faculty of Mechanical Engineering, Chemnitz University of Technology, 09111 Chemnitz, Germany

**Keywords:** total hip arthroplasty, insertion, acetabulum, modular hip implant, ceramic liner, surgical instrument, malseating

## Abstract

Modern hip implants have a modular design. In case of wear or other damage it allows surgeons to change the tribological partners, i.e., the acetabular liner and femoral ball. In both revision and primary surgery, the secure joining of the implant components is important for the success of the operation. The two components, namely the ceramic liner and hip cup, are connected via a conical press connection and should be concentrically aligned to avoid chipping. Malseated liners can reduce the life span in situ. The amount of the joining force, which is usually applied via a hammer, depends on the surgeon. In this study, an alternative joining method for acetabular ceramic liners in hip cups was investigated, which intends to make the process more reproducible and thus safer. For this purpose, a handpiece was used to apply a defined force impulse of 4 kN. For the concentric alignment of a ceramic liner in the hip cup, an adapter was developed based on findings via a qualitative finite element (FE) analysis. Insertion and pushout tests of the acetabular cup–liner connection were performed in the laboratory with the new instrument (handpiece with the connected adapter) to evaluate the functionality of the instrument and the reproducibility of the new insertion method. For comparison, liners and acetabular cups were joined using a testing machine according to the standard. The presented results demonstrate the technical proof-of-concept of the new joining method under laboratory conditions. They meet the acceptance criteria of established manufacturers, which proves the equivalency to proven methods for joining modular implant components. To verify the improvement of the new joining method compared to the conventionally used joining method, an application-oriented study with different surgeons and the new joining instrument under clinical conditions is necessary.

## 1. Introduction

Due to aging societies [1], increasing life expectancy [2] and increasing demands on the quality of life in old age, implants are becoming more complex and are expected to achieve a longer life span in situ. In 2021, 158,690 initial implantations and 17,752 revision surgeries (in total 176,442 operations) were reported to the German Arthroplasty Registry (EPRD) for hip replacement in Germany [3]. According to the Global Hip Replacement Market Report 2021–2027, the market will increase by 6.77% annually until 2027 [4].

Modern hip implants are modular, i.e., the joint-forming implant component consists of at least three separate parts. Through revision surgery, friction partners such as liner or head can thus be changed without having to replace the implant components located in the bone (cup, hip stem), which preserves the remaining bone material. The modular implant components are connected to the elements anchored in the bone via a conical press connection (radial press fit). The acetabular liner (e.g., made of ceramic) is joined by an impactor instrument and several impacts [5,6,7]. The intraoperative joining of the modular components is usually not reproducible, i.e., the joining force and alignment of the instrument depends on the surgeons. In a study by Fritsche et al., the impact forces to create a press fit between the liner and hip cup varied between 1 and 8.9 kN [8].

Metal abrasion at the junctions of modular implants is assumed to be a reason for failure and associated revision surgery in total hip arthroplasties [9,10,11]. Although the ceramic–ceramic bearing couple shows lower wear rates [12], a malseated ceramic liner in the metal cup can lead to fretting, metallosis and, thus, the loosening of the implant [13]. Furthermore, jamming, point loads or chipping and, in rare cases, fractures of ceramic liner [14,15,16,17,18] may occur. The literature indicates that between 7.2% and 16.4% of ceramic liners are malseated [19,20,21]. Although malseated liners do not always negatively affect the success of the surgery, the use of careful surgical technique and the concentric alignment of the liner in the cup is recommended [13,15,22,23,24]. The joining process and the associated surgical technique are the most significant factors that can be influenced to ensure a secure press fit connection [24,25].

McAuley et al. investigated the factors influencing fractures in ceramic liners and were able to simulate through practical tests that misaligned ceramic liners increase the risk of fracture. At the same time, the quality of the connection is improved via intense and repeated hammering [25].

Previous studies describe the status of the joining methods for implant components and problems resulting from misaligned liners. They require careful surgical technique, but current joining methods allow for mistakes. A reproducible and controlled insertion method of hip implant components can lower failure rates due to metal abrasion (by reducing relative motions due to insufficient joining forces), chipping or ceramic fractures. Therefore, this work aims to test the technical feasibility of a reproducible insertion method of modular acetabular ceramic liner using a novel instrument. Our hypothesis is that joining over the edge surface of the line is as good as established joining methods. An adapter developed for this instrument aligns the ceramic liner axially. The impaction force is applied via the edge of the liner using an commercial handpiece with a reproducible force impulse of 4 kN [26]. The evaluation of the feasibility is carried out on the one hand via finite element (FE) analysis. On the other hand, laboratory tests are carried out to determine the push-out forces of joined implant components according to ASTM F1820-13 “Standard Test Method for Determining the Forces for Disassembly of Modular Acetabular Devices” and compare them to the internal acceptance criteria of implant manufacturers on the market.

## 2. Materials and Methods

### 2.1. Concept of the Instrument

**Figure 1:**
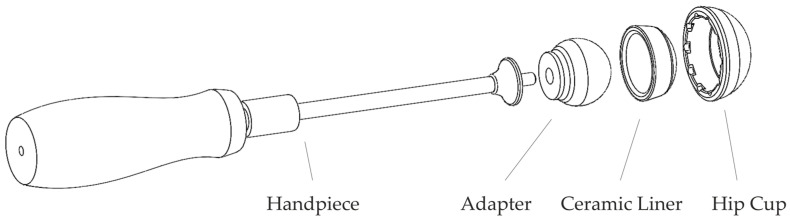
Instrument assembly.

In order to implement the new reproducible insertion method, a new instrument has been developed in a collaborative research project called “Smart-I” together with manufacturers of implants and surgical instruments (see the Acknowledgments). The instrument consists of a handpiece designed by endocon GmbH (Wiesenbach, Germany), which is already available on the market under the name safeConnect^®^ [26]. It applies a reproducible, defined impact force under pulse control and is dimensioned at 4 kN. On the handpiece, there is an exchangeable adapter, which forms the interface to the acetabular ceramic liner and has been designed according to the requirements of the FE analysis. Figure 1 shows the general structure of currently used instruments without concentric alignment through the adapter and the two joining components, i.e., the acetabular cup and the liner. This general structure was retained during development.

### 2.2. FE Analysis

For the concept design of the instrument’s adapter, the first step was examining the conical locking mechanism between the cup and liner using static mechanical FE analysis with the Ansys 2021 R2 software (Ansys Inc., Canonsburg, PA, USA). This is a numerical method used for different physical tasks like strength and deformation testing. For this purpose, the force application angle and location were varied.

The joining of the liner and acetabular cup is based on a friction-locked connection, which is realized via a conical press fit. The liner is inserted into the cup with force pulses of hammer impacts. Currently, available instruments, transfer force to the concave surface inside the liner via a ball [5,6,7,26]. This method is the currently used conventional insertion method for modular acetabular ceramic liners and hip cups. Due to manufacturing inaccuracies, there are often only point or line loads on the joining surfaces which, due to the resilience of the materials, is often distributed over a small area. In addition, the instrument with a ball head adapter allows the tilting of the instrument during joining, which results in decentralized force application in the liner.

With the help of FE analysis, the conventional joining method was analyzed for varying force introduction angles and locations with an impaction force of 4 kN. In addition to the axial load introduction (0°) and two tilted introduction angles (14.5° and 29°) were studied to quantify the influence of the force angle on the stress distribution in the ceramic liner and the hip cup and the resulting push-out forces.

In comparison, an alternative joining method was analyzed in which the force is not introduced to the liner’s curved surface but via the planar upper edge surface of the liner. The FE analysis is intended to verify whether the applied impaction loads are distributed more evenly in the liner through this joining method and whether occurring regular forces are distributed more homogeneously in the joining interface between the liner and cup. In addition, a uniformly applied load at the liner edge could counteract tilting. The influence of the force application location and its direction were investigated using the following model:

Material: The material models for the ceramic liner made of ATZ (Alumina Toughened Zirconia Ceramic “ceramys” [27]) and the hip cup were assumed to be isotropic. On the liner side, a linear elastic model approach was chosen with a modulus of elasticity of 260 GPa and a Poisson’s ratio of 0.22 [28]. This assumption was made because the ceramic liner does not deform plastically due to its significantly higher modulus of elasticity than the hip cup’s material. Ti6Al4V was specified as the material for the cup. The Young’s modulus is 120 GPa and the Poisson’s ratio is 0.3. For the bilinear isotropic strain hardening, a tangent modulus of 1 MPa was specified from a yield strength of 860 Mpa [29].

Contact conditions: The contact conditions for joining the liner and hip cup were realized via frictional contact. The friction coefficient was calculated according to Equations (1)–(3) [30] based on internal impaction and push-out force data from Mathys Orthopädie GmbH (Mörsdorf, Germany).

(1) $$F_{N}=\frac{F_{ax}}{sin\left(\alpha \right)}$$

(2) $$F_{L,ax}=F_{N}\cdot cos(\alpha )\cdot (\mu_{ax}-tan\;\left(\alpha \right))$$

(3) $$\mu_{ax}=\frac{F_{L,ax}}{F_{N}\cdot cos(\alpha )}+tan(\alpha )$$

The friction value was determined from the axial impaction force $F_{ax}$, the normal force $F_{N}$ resulting from the cone angle α, according to Equation (1). Subsequently, Equation (2) was converted to $\mu_{ax}$ via the normal force and the experimentally determined axial push-out force $F_{L,ax}$ (Equation (3)). This resulted in a friction value of 0.24 for a cone angle of 9.44°. The Augmented Lagrangian method was used to formulate the contact equations. The contact surfaces were not trimmed and were adjusted to contact at the beginning of the calculations. Furthermore, the contact stiffness was updated after each iteration and was set to 0.2 as a compromise between accuracy and convergence behavior to improve the convergence behavior of the simulations. Furthermore, an automatic bisection was considered an automatic time step control.

Mesh: Tetrahedral elements with a quadratic shape function were chosen for the calculation. A mesh convergence study was performed, where the refinement of the mesh size from 1 to 0.5 mm resulted in only a 0.11% change of the maximum stress in the liner. This is the reason why a mesh size of 1 mm was chosen. The entire calculation mesh contains 93,380 elements and 153,210 nodes with an average skewness of 0.36. Identical contact meshing was carried out in the contact area. The mesh settings were set globally.

**Figure 2:**
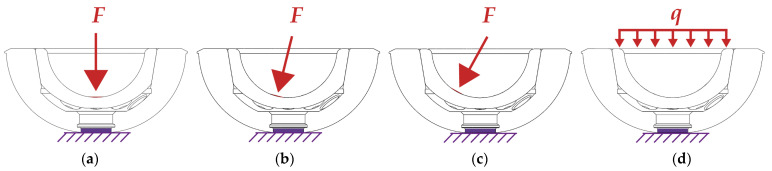
Force application. (**a**) Axial, conventional method (0°); (**b**) conventional method under 14.5°; (**c**) conventional method under 29°; (**d**) alternative joining method on the upper surface of the liner.

Initial and boundary conditions: For the force application (see Figure 2), the surfaces of the geometry models of the liner were adapted in order to apply the force with a ball head on a small surface load. Therefore, the surface for the force application was set to 19.6 mm^2^ (radius of 2.5 mm). For the alternative insertion method, a constant surface load of 4 kN was applied to the upper side of the liner. The hip cup was fixed at the centric hole in the bottom.

**Figure 3:**
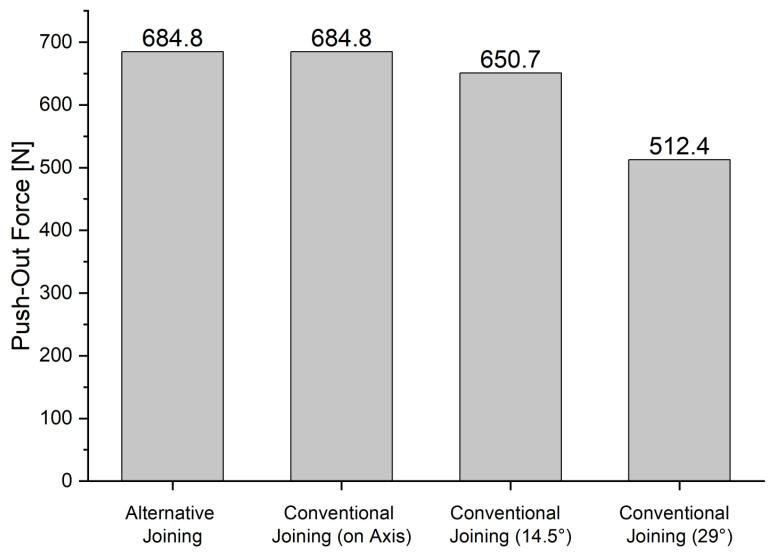
Relative comparison of push-out forces for the different joining angles and methods.

Findings: To evaluate the joint connections of the different load application angles and the new load application method, the push-out force $F_{L,ax}$ (Equation (2)) and the von Mises equivalent stress of the different versions were compared with each other. The push-out force of 684.8 N for axially conventionally joined liners equals the push-out force of the alternative joining method (see Figure 3). With an oblique force insertion of 14.5°, a reduction in the push-out force to 650.7 N was calculated, which is 4.98% smaller. In contrast, the resulting push-out force decreases significantly with increased force application angle. The resulting push-out force at an application angle of 29 is 512.4 N and thus is 25.2% smaller than the push-out force with axial joining.

**Figure 4:**
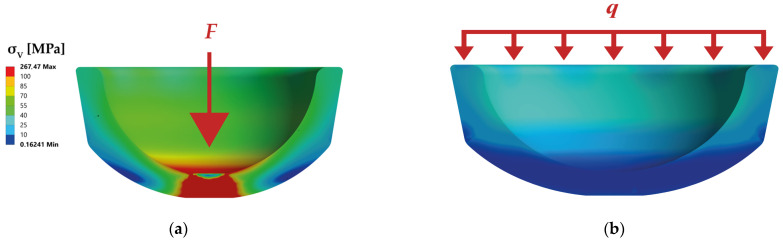
Comparison of stress distribution of the liner for conventional method (**a**) and alternative joining method (**b**).

Furthermore, the loads on the liner during conventional joining are very high due to the small force application area (see Figure 4). With axial, conventional joining, maximum equivalent stresses in the liner of 267.5 MPa were calculated, whereas the equivalent stresses in the alternative joining method are only 39.8 MPa. Thus, the maximum stress on the liner can be reduced by 85 % by distributing the impact force over the line’s edge. As a result, the alternative joining method protects the liner with equivalent push-out forces.

The preliminary numerical investigation shows the importance of axial alignment of the instrument, as an angled load application decreases the resulting push-out force. Furthermore, the load on the liner can be significantly improved by shifting the force applied to the edge surface of the liner. However, according to the current state of the art and to the authors’ knowledge, there are no adapters for pulse-controlled surgical tools such as the safeConnect^®^ [26] with axial alignment and load application via the edge of the liner for the reproducible joining of the hip cup and liner. Based on this numerical preliminary investigation, an adapter for applying axial impact forces via the edge of the liner is designed and experimental trials are planned to test the feasibility of the alternative joining method under laboratory conditions.

The simulations showed that the force application via the edge surface of the insert achieves equivalent push-out forces with significantly lower loading of the ceramic insert. However, these are not validated models, which were only used for qualitative load analysis of the joining process. Further experimental validation would be required to use the models for the component design of the cup or insert.

### 2.3. Impacting and Push-Out Tests

In order to test the developed alternative joining method, i.e., the adapter for its suitability for joining ceramic liners in hip cups, these components were joined manually with the new instrument and compared using the testing machine according to ASTM F1820-13. It is standard procedure to measure the attachment strength between the hip cup and liner, specifying the test setup for axial disassembly or the test speed. To evaluate the achieved connection quality, the respective push-out forces according to ASTM F1820-13 were determined and compared. Due to a lack of samples, the number of specimen deviates from the standard. For the two test series (joining via instrument and via testing machine) six repetitions were performed, with one component pair available per series. Due to the expected plastic deformation of the components during the first insertion process, which leads to higher push-out forces compared to the following tests, the first test data were not included in the evaluation. Five evaluable test datasets were used to calculate the mean value of the resulting push-out force.

An acetabular hip cup (Multicup II, Merete GmbH, Berlin, Germany) with a cup diameter of 58 mm manufactured by Aristotech Industries GmbH (Luckenwalde, Germany) and a 32-ceramic liner (ceramys) by Mathys Orthopädie GmbH (Mörsdorf, Germany) were used.

**Figure 5:**
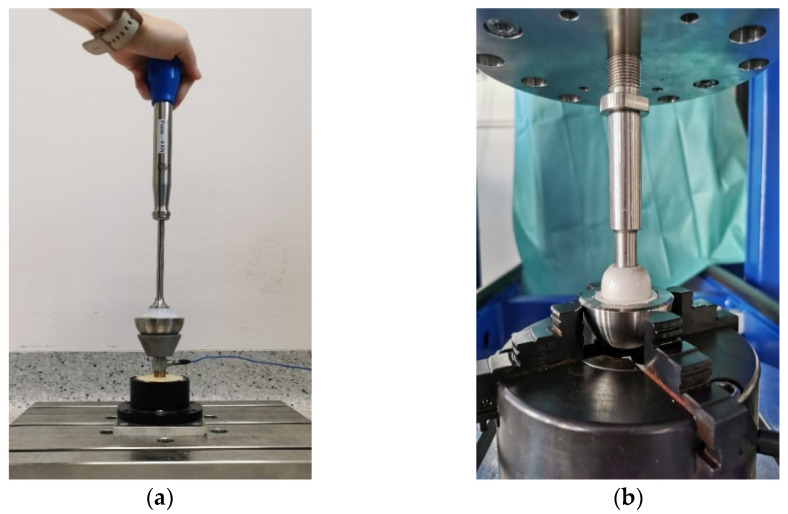
Test setup for insertion with an instrument (**a**)—note that the impulse sensor was not used in the current study—and insertion with the testing machine (**b**).

When joining with the instrument, the force was transferred to the components via an impulse triggered in the handpiece (see Figure 5a). For this purpose, the cup was placed in a holding device, the contact surface of which corresponded to the outer contour of the cup. For impacting and push-out with the testing machine (tensile/compression/torsion testing machine from DYNA-MESS Prüfsysteme GmbH, Aachen, Germany), the test speeds were selected according to the test standard ASTM F1820-13. The liner was pressed in at 0.04 mm/s (see Figure 5b) and pushed out at 5.1 cm/min.

## 3. Results

### 3.1. Technical Realization of the Adapter

An adapter was developed for the instrument used to join the liner and cup, which had the following functions:

1. Straight pick up of the liner;
2. Hold the liner;
3. Align the instrument to the hip cup;
4. Transmit forces.

**Figure 6:**
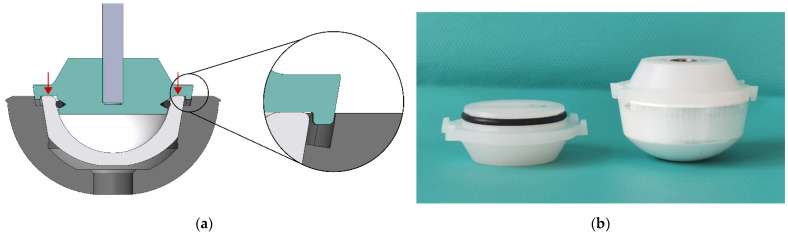
Adapter: (**a**) enlargement of the adapters lugs and the recess in the hip cup; force transmission highlighted with red arrows. (**b**) Manufactured adapter with (**right**) and without liner (**left**).

The implemented preferred concept for the adapter introduces the force via the upper liner edge. The liner is held on the instrument during the implantation process by the clamping force of an upset O-ring on the concave inner surface of the liner. The liner is aligned straight to the instrument by the contact surface on the liner edge. The developed adapter enables one-handed placement in the cup during surgery. For axial alignment, the adapter is guided straight into recesses in the hip cup with the help of lugs on the liner without retaining the hip cup (see Figure 6a). Thus, the impact force is primarily used to connect the two components.

PPSU (TECASON P MT ivory, Ensinger GmbH, Nufringen, Germany) was used as material for the adapter. It is a high-performance plastic often used for surgical instruments, that needs to be resistant to typical sterilization methods. The adapter was manufactured by Mathys Orthopädie GmbH (Mörsdorf, Germany) with the close adjustment of the manufacturing tolerances. It consists of three parts: the main body with a pressed-in threaded sleeve and the O-ring (see Figure 6b).

### 3.2. Evaluation of the Alternative Joining Method with Insertion and Push-Out Tests

The measured push-out forces for removing the liner from the cup, joined by the instrument and the testing machine, are listed in Table 1.

**Figure 7:**
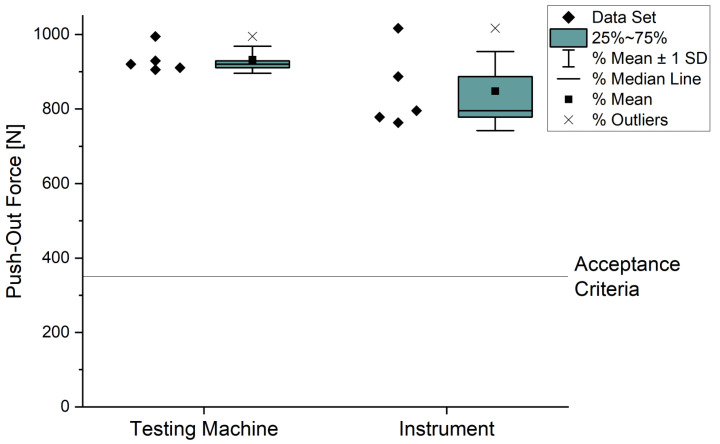
Push-out forces of components joined by testing machine and instrument.

The average push-out forces ($F_{I}=848.06\;N;F_{TM}=932.27\;N$) differ only by approximately 80 N. The standard deviation for the push-out force of the instrument is $\sigma_{I}\approx 106\;N$ and for the testing machine is $\sigma_{TM}\approx 36\;N$. The statistical spread of the push-out forces of the components joined by the instrument is higher than those joined by the testing machine. The difference between the two average push-out forces is not significant for a significance level of 0.05 and a *p*-value of 0.15 (*t*(4.92) = −1.68). However, all tests fulfill the internal acceptance criteria of Mathys Orthopädie GmbH (Mörsdorf, Germany) with 350 N (see Figure 7). This is an internal acceptance criteria, which is based on test results of Steinhauser et al. [31] and multiplied with a safety factor of 1.5. CeramTec’s acceptance criteria (approx. 225 N [25]), which is below our requirement for the connection.

Using the mean push-out forces and an impaction force of 4 kN, the quotient (push-out force divided by impaction force) is 0.233 for the testing machine and 0.212 for the instrument.

## 4. Discussion

The quality of the connections of the hip implant components significantly influences the success and life span in situ of operative care with total hip arthroplasty. The push-out force of the modular components is influenced by the surgical technique, like the applied impacting force [24,25]. The applied impacting forces vary from surgeon to surgeon.

In a study by Lee et al. [32], ceramic liners with two different taper angles (10° and 18°) were manually inserted by three surgeons. They determined the push-out forces and the malseating rates of the ceramic liners with the result of higher push-out forces for a 10° taper than those in 18°. The authors recommend careful handling to avoid malseating when joining the ceramic liner and the acetabular cup with smaller angles. In our study, the component taper angle was 9.44°, which is why a straight alignment is desirable.

Therefore, this work aimed to evaluate the feasibility of a new insertion method with reproducible forces to achieve the press fit connection of the ceramic liner and acetabular cup. For this purpose, these components were joined using an instrument consisting of a handpiece (safeConnect^®^) already on the market and a newly developed adapter. The push-out forces were compared to the components joined according to ASTM F1820-13. The push-out forces for the joined components were above the acceptance criteria. The mean values for both joint connections do not differ significantly when comparing the push-out forces of the components joined with the instrument and the components joined with the testing machine. It is likely that the new instrument and thus the new joining method over the liners edge had no significant influence on the connection. It is equal to proven methods.

The push-out forces on the testing machine were higher by 80 N on average. One reason for the slightly increased forces could be the different methods of force application. The testing machine applies the force quasi-statically, which means that a target force of 4 kN has to be reached and held for a short time. If, for example, a component slips (stick–slip effect), the testing machine ensures that the target force is still reached. The instrument, on the other hand, triggers an impulse once. Should the components slip during the insertion process, this is not compensated for by readjustment. Furthermore, the testing machine almost ideally applies the forces straight to the components. In contrast, the instrument may have a slight angular misalignment of the axes due to the clearance fits chosen for guiding the lugs of the adapter in the recesses of the cup. This would also explain the greater variation in the values of the push-out forces.

A greater number of samples in a larger study would statistically validate this investigation. This data could be used to validate the FE analysis, because the analysis performed in this study was not validated through experiments. But the quantitative statements made were sufficient for the design of the adapter and therefore sufficient for this investigation. Another limitation is that the tests were carried out under almost ideal but simplified conditions in the laboratory. Further studies should still verify the improvement compared to currently used instruments and under real conditions in the operating room.

The application of force via impulse, the force level, and the surrounding tissue influences the achieved push-out forces. Although ceramic liner fractures are well known, they are rarely reported in the literature. But there are studies that describe the influencing factors when joining the hip stem and ceramic head. As this connection is also a conical press fit, the dependencies could be relevant for the joining of ceramic liner and acetabular cup as well. From a technical point of view, the connection mainly differs in the length of the cone, which tends to have a positive effect on self-centering and is therefore less conducive to tilting.

We compared the push-out forces of impulsively joined components with quasistatically joined implant components. In a study by Wade et al. [33], six insertion methods for joining the ball and hip socket were compared concerning the press fit achieved at the joining partners. The best results were achieved with a quasi-static method joined concentrically to the loading direction. The self-alignment of the components did not work for joining via hammering if the ball was initially placed incorrectly. This could be another reason why the push-out forces achieved with the testing machine were higher than those achieved with the instrument. It would be interesting to compare the push-out forces of the developed instrument with a test set-up in the laboratory [34], where joining is also carried out through impulse.

The influence of the surrounding tissue and the force level were not considered in this study. Krull et al. [35] showed that an increase in impact energy leads to an elevation of the resulting forces on the surrounding tissue/bone of the patient. However, if the stiffness of the impact tip of the instrument is increased, this does not influence the forces transmitted to the patient; in turn, the pull-off force of the joined components of the implants increases. Furthermore, it was shown in the laboratory via a standard test procedure that the patient’s flexibility has no influence on the resulting forces of the joined taper joint [34]. Wendler et al. analyzed the different impact forces on the ball/stem side of 31 surgeons on a replacement model. They concluded that the achieved joining force depends significantly on the damping behavior of the tissue surrounding the implant. Due to the large variance and the too-low joining forces (according to the literature, the impact force should be 4 kN [34,35,36,37,38]), they also recommend a standardization of the impaction using a suitable instrument [39]. The optimal impact force and the influence of the amount of this force should therefore also be further investigated regarding the insertion of the liner in the cup. The selected material, the geometric design and the adapter’s stiffness of the instrument significantly influence the achieved joining force and thus also the push-out force of the conical press connection of hip implant components.

## 5. Conclusions

Simulations showed that load application via the liner edge is gentler on the ceramic than conventional load application via the inner concave liner surface. As a result, an adapter was developed which, in addition to the alternative load application, also enables axial guidance and thus straight load application. The small deviations of the tests with a testing machine and the achievement of the acceptance criteria of established implant manufacturers show that when comparing the push-out forces, the new method is equivalent to proven insertion methods. The functional proof-of-concept of the instrument, including the adapter, by joining the hip implant component ceramic liner to the cup was demonstrated.

In future research, mechanical tests should be carried out with a larger number of components to validate the FE analyses performed. These tests can answer the question as to whether this new insertion method and the developed instrument could lead to less chipping or fewer ceramic fractures.

To show the clinical relevance and the benefit of the new instrument, a comparison with conventionally used instruments with qualified personnel from a clinic is still outstanding. The influence of the bony environment on the achieved joining force or the access routes has not yet been sufficiently investigated, nor have the advantages for liners made of other materials, like PE. All patients who need a hip implant benefit from safe joining method and a longer life span in situ.

## Figures and Tables

**Table 1:** Push-Out forces.

Trial Number	Push-Out Force [N] Joined by	
	Testing Machine $F_{TM}$	Instrument $F_{I}$
1	929.45	886.87
2	920.38	1016.72
3	910.99	795.31
4	994.86	763.33
5	905.65	778.06
Mean Value	932.27	848.06

## Data Availability

The data presented in this study are available on request from the corresponding author.
